# Local climate, air quality and leaf litter cover shape foliar fungal communities on an urban tree

**DOI:** 10.1007/s13280-024-02041-4

**Published:** 2024-06-13

**Authors:** Maria Faticov, Jorge H. Amorim, Ahmed Abdelfattah, Laura J. A. van Dijk, Ana Cristina Carvalho, Isabelle Laforest-Lapointe, Ayco J. M. Tack

**Affiliations:** 1 Department of Ecology, Environment and Plant Sciences, Stockholm University, Frescativägen 40, 114 18 Stockholm, Sweden; 2 Département de Biologie, Université de Sherbrooke, 2500, boul. de l’Université, J1K 2R, Sherbrooke, QC Canada; 3 Swedish Meteorological and Hydrological Institute (SMHI), Folkborgsvägen 17, Norrköping, Sweden; 4 Leibniz Institute for Agricultural Engineering and Bioeconomy (ATB), Max-Eyth-Allee 100, 14469 Potsdam, Germany

**Keywords:** Air quality, Fungal communities, Fungal guilds, Leaf litter cover, Local climate, Urban trees

## Abstract

Foliar fungi on urban trees are important for tree health, biodiversity and ecosystem functioning. Yet, we lack insights into how urbanization influences foliar fungal communities. We created detailed maps of Stockholm region’s climate and air quality and characterized foliar fungi from mature oaks (*Quercus robur*) across climatic, air quality and local habitat gradients. Fungal richness was higher in locations with high growing season relative humidity, and fungal community composition was structured by growing season maximum temperature, NO_2_ concentration and leaf litter cover. The relative abundance of mycoparasites and endophytes increased with temperature. The relative abundance of pathogens was lowest with high concentrations of NO_2_ and particulate matter (PM_2.5_), while saprotrophs increased with leaf litter cover. Our findings show that urbanization influences foliar fungi, providing insights for developing management guidelines to promote tree health, prevent disease outbreaks and maintain biodiversity within urban landscapes.

**Supplementary Information:**

The online version contains supplementary material available at 10.1007/s13280-024-02041-4.

## Introduction

In urban environments, trees play a vital role in providing ecosystem services, such as local climate regulation, urban biodiversity maintenance and enhancing human wellness (Brack 2002; Jennings and Gaither 2015; Turner-Skoff and Cavender 2019). However, these functions are not solely attributable to trees themselves, but are linked to their associated microbial communities. Microorganisms, including fungi, contribute to tree host stress tolerance, growth and resource acquisition, but can also cause diseases (Newbound et al. 2010; Abrego et al. 2020). Fungi can also degrade air pollutants through oxidation mechanisms, participate in carbon sequestration and cause allergies in humans (Weyens et al. 2009; Kasprzyk et al. 2021). While fungi associated with tree leaves are an important part of the urban tree microbiome, we lack an understanding of how these communities are influenced by urbanization (but see, Jumpponen and Jones 2010). This knowledge gap hinders our understanding of the relationships between urbanization, fungal communities, and the overall functioning of urban trees.

The urban microclimate can have a significant impact on the diversity and community structure of foliar fungi. Cities tend to be warmer than surrounding rural areas due to the urban heat island effect, which can create climatic conditions that are more favourable for certain groups of fungi (Velásquez et al. 2018). For example, fungal pathogens may favor trees that grow in warmer and drier locations of the cities (Tubby and Webber 2010; Kimic et al. 2023). Similarly, saprotrophs—fungi that play a role in litter decomposition—may also prefer warmer (but humid) urban locations (Morrison et al. 2019). Temperature and humidity can influence the dynamics of foliar fungi directly, by affecting their germination and growth, but also indirectly by inducing physiological changes in host trees. For example, warmer temperatures and droughts can increase the susceptibility of host trees to fungal pathogens and the susceptibility of fungi to mycoparasites (Hossain et al. 2019; Desaint et al. 2021). Overall, understanding the relationship between local climate and the dynamics of foliar fungi is crucial for managing urban trees and mitigating potential negative impacts of climate change on urban tree health.

Apart from climate, other factors related to urbanization can influence fungal communities on leaves. One such factor is air quality, which can affect the diversity, abundance, and community structure of foliar fungi. For example, airborne fungal groups, such as endophytic fungi *Penicillium* were shown to increase in response to nitrogen dioxide (NO_2_) concentrations, while a sooty mold, *Aureobasidium* spp., decreased in response to NO_2_ (Pyrri and Kapsanaki-Gotsi 2017). However, while fungal groups have shown varied responses to air pollutants, the overall impact on fungal community structure remains unclear. Studies exploring the effects of air pollution on fungal community structure have yielded conflicting results, with several of them thus far demonstrating a positive, negative or no effect of air pollution on fungal richness, evenness, abundance and community composition (Bearchell et al. 2005; Cao et al. 2014; Du et al. 2018; Fan et al. 2019). Several mechanisms can explain the effect of air pollutants on fungal communities. First, air pollutants, such as nitrogen dioxide and ozone can inhibit fungal growth, damage cellular structures and interfere with the metabolic processes (Li et al. 2022). Second, they can weaken plant defenses, making them more susceptible to fungal attacks. For example, pollutants can damage the leaf cuticle and impair the production of antimicrobial compounds (Blande et al. 2014). Finally, certain air pollutants, such as particulate matter with an aerodynamic diameter smaller than 2.5 μm (PM_2.5_), can improve fungal growth by providing additional nutrient sources (Weyens et al. 2015). Overall, further research is necessary to understand the impact of air pollution for foliar fungi in urban environments.

Heterogeneous urban environments offer a large variety of habitat types for plants to grow, including parks, gardens and built areas. Those habitats vary not only in climatic conditions and levels of air pollution, but also in terms of light availability, which is crucial for the development of plants and their associated fungi (Sabburg et al. 2015). Urban management practices can also influence fungal communities on trees. Leaf litter removal, for example, can impact the diversity and community structure of foliar fungi, as many fungi use leaf litter to overwinter and as a substrate for sporulation (Irga et al. 2016). Leaf litter cover may have varying effects on specific fungal guilds; with, for example, fungal saprotrophs favoring locations with higher leaf litter coverage (Marañón-Jiménez et al. 2021). Similarly, many fungal pathogens were found to overwinter in the leaf litter under infected trees, poised to recolonize trees upon the onset of spring (Jain et al. 2019). Besides local habitat factors, the spatial connectivity of plants in urban environments could also affect the distribution of fungal communities. For example, trees that grow in close proximity with conspecifics might have different fungal communities compared to trees that are less connected or isolated (van Dijk et al. 2022; Faticov et al. 2023). Hence, local climate, air quality levels, habitat factors and host connectivity can collectively shape the diversity and community structure of tree fungal communities in urban environments. By unraveling these complex relationships, we can gain insights into the management of tree-associated microorganisms, such as pathogens.

**Fig. 1:**
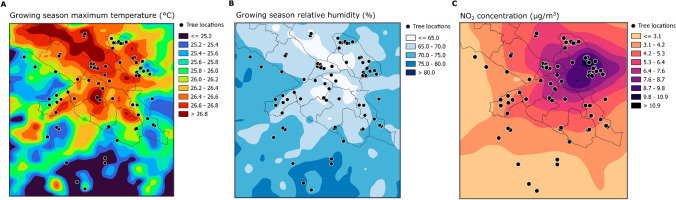
Location of the 79 oak trees (*Quercus robur*) in Stockholm region, Sweden from which we sampled the foliar fungal community. Locations are superimposed on a background map with local **a** growing season maximum temperature, **b** growing season relative humidity, and **c** NO_2_ concentrations

We investigated the effects of local climate, air quality, habitat factors and host connectivity on foliar fungal communities. For this, we first developed high-resolution climatic and air-pollutant maps for Stockholm region, Sweden (Fig. 1), and subsequently sampled foliar fungal communities of 79 pedunculate oaks (*Quercus robur L.*) across this region (Fig. 1). Specifically, we aimed to answer the following questions:

1. What is the relative importance of local climate, air quality, habitat factors and host connectivity on foliar fungal richness, evenness, and community composition?
2. What is the relative importance of local climate, air quality, habitat factors and host connectivity on the relative abundance of functional guilds? Which taxa are most sensitive to urbanization?

Our detailed predictions are presented in Table S1 in the Supplementary information.

## Materials and methods

### Study system

In this study, we focused on the pedunculate oak, *Quercus robur,* which is one of the dominant deciduous tree species in Europe, where it is of great ecological, economic and social importance (Eaton et al. 2016; Mölder et al. 2019). The pedunculate oak grows on a wide range of soil types in forests, wooded pastures and agricultural landscapes, and reaches its northern limit in central Sweden (Stenberg and Mossberg 2003). Notably, pedunculate oaks are also commonly found in cities and urban parks across Europe, including Sweden (Sjöman and Slagstedt 2015; Willis and Petrokofsky 2017). *Q. robur* has a long natural life span and offers a habitat to a high diversity of species, including fungi (Jumpponen and Jones 2009; Faticov et al. 2021; van Dijk et al. 2022).

### Study design

To link the effects of local climate, air quality, habitat factors and host connectivity to the tree leaf microbiome, we sampled 79 mature oaks (*Quercus robur*; five leaves from each tree) across a climatic and air quality gradient within Stockholm, Sweden (Fig. 1). Leaf sampling was conducted from branches not higher than 2 m above the ground. To minimize the effect of differences in leaf age, we conducted leaf sampling between July 1st and 14th, 2021, and we only collected fully developed leaves (Gaytán et al. 2022). Samples were collected in separate Ziploc bags and stored in a cooler box during transportation. At the laboratory, we used a metal corer to punch four leaf disks with a diameter of 5 mm from each side of the midrib of each of the five replicate leaves per tree. Hence, each sample consisted of 40 pooled leaf discs thus standardizing sample size. All processing was done in a laminar flow hood, and the metal corer was sterilized after processing each sample with 95% ethanol and flaming over a Bunsen burner. Samples were freeze dried and ground into fine powder using a bead mill (TissueLyser II; Qiagen) before DNA extraction.

### Molecular methods

DNA was extracted from 10 mg of leaf material using the NucleoSpin Plant II kit (Machery-Nagel, Düren, Germany) following the standard protocol. To characterize foliar fungal communities, we used primers targeting the ITS2 region (Schoch et al. 2012). We used the forward primer fITS7 (Ihrmark et al. 2012) and reverse primer ITS4 (White et al. 1990). For DNA amplification, PCR reactions (one reaction per sample) were run in volume of 25 µL and the reaction mixtures were prepared using Kapa HiFi Mastermix (Kapa Biosystems, Woburn, MA, USA). We ran one PCR reaction for each sample. The final library was sequenced at SciLifeLab/NGI (Solna, Sweden) on Illumina MiSeq (Illumina Inc., San Diego, CA, USA) with 2 × 300 bp reads. For more details on library preparation, including cycle number and cleaning procedures, see Text S1.

In total, we obtained 9,920,057 sequences from 79 samples after quality filtering and removal of sequences that appeared in negative controls. The sequences were clustered into 5092 amplicon sequence variants (ASVs) using DADA2 (Callahan et al. 2016). On average, fungal communities were represented by 117,993 reads per sample. To exclude any spurious ASVs that might have been created by PCR or sequencing errors, we filtered the ASVs to remove those that were represented by fewer than 20 sequences using the function *prune_samples* in the *phyloseq* package (McMurdie and Holmes 2013). When calculating fungal species richness (number of ASVs per sample) and Pielou’s evenness (Pielou 1966), we accounted for uneven sequencing depth by rarefying each sample to 2000 reads. For community composition analyzes, we used MetagenomeSeq’s cumulative sum scaling (CSS) as a method to account for uneven sequencing depth (Paulson et al. 2013). To conduct guild analysis, we manually assigned functional traits to ASVs based on their taxonomic rank at the genus level using the *FungalTraits* database (Põlme et al. 2020). We assigned ASVs to a total of seven functional guilds: (i) sooty molds, (ii) endophytes, (iii) saprotrophs, (iv) pathogens, (v) mycoparasites, (vi) other (which included animal and lichen parasites, lichenised fungi and a few ASVs assigned to epiphytes), and (vii) unknown fungi (see Table S2 and Supplementary Data 1 for details). We then calculated the relative abundance of each fungal guild (i.e., the summed number of reads of all ASVs in each guild out of the total number of reads) for each sample.

### Climate, air quality, habitat and host connectivity data

The high-resolution data describing the climate and air quality of the city of Stockholm were created within the Copernicus Climate Change Service (Amorim et al. 2020; Gidhagen et al. 2020). As an alternative to model a long climate time series (typically 30 years), leading to a very high computational cost, this proof-of-concept considered 5 years in the recent past (2006, 2007, 2012, 2013 and 2014) that represent different climate conditions. By comparison with WMO’s Climatological Standard Normals, these years can be classified in terms of local temperature/precipitation anomalies as follows: warm/wet, warm/dry, normal-to-warm/wet, warm/dry and warm/normal-to-wet (see Table S3).

The 1 km × 1 km resolution data was produced in Urban SIS with a dynamical downscaling technique that involved the convection-permitting limited-area numerical weather prediction (NWP) system HARMONIE-AROME (cycle 40 h1.1, Bengtsson et al., 2017) and the multi-scale atmospheric transport and chemistry (MATCH) model (Robertson et al. 1999). The atmospheric interactions between surface and atmosphere, a fundamental aspect within the analysis carried out in this paper, were computed using the SURFEX model (version 7.3; Masson et al. 2013) coupled online to the NWP model. SURFEX divides the surface in tiles and patches, making it possible to account for sub-grid spatial heterogeneity, of prime importance in urban areas. For this purpose, a high-resolution 300 m × 300 m physiography database was used that combined different open-access products for the highest detail possible. Meteorological lateral boundary conditions were retrieved from the UERRA-ALADIN reanalysis (Ridal et al. 2016) on a 11 km × 11 km grid spacing. The surface data assimilation included observations from surface synoptic and airport stations of near-surface temperature, relative humidity and snow water equivalent provided by the Meteorological Archival and Retrieval System (MARS) at the European Center for Medium-range Weather Forecasts (ECMWF).

The chemical transport model MATCH includes enhanced features, such as deposition, advection, vertical diffusion, various chemistry schemes and data assimilation (Robertson et al. 1999). The chemical mechanism and the aerosol module are based on those implemented in EMEP MSC-W model with adaptations on isoprene chemistry and on the volatile secondary organic aerosols. In the framework of Urban SIS, the MATCH model was applied following a downscaling approach similar to the NWP. The inner computational domain covering Stockholm city was simulated using the meteorological fields provided by the 1 km resolution NWP runs and considering high-resolution air pollutant emissions data. To have a better description of the long-range transport and background concentrations, this domain had as boundary conditions the chemical species concentrations calculated over a pan-European region at the lower resolution of 0.2° × 0.2°, forced by the UERRA reanalysis, and the anthropogenic emissions taken from the MACC inventory (Kuenen et al. 2014).

For each of 79 oak trees, average monthly time series covering the 5 years, were extracted from detailed urban climatic and pollutant maps. As correlations of climate and air quality parameters across years were very strong (Pearson’s *r* > 0.9 for all among-year correlations, and with very few rank reversals), we used the 5-year averages for further analyzes. The parameters selected for the analysis were air temperature and air humidity, and the ground level concentrations of NO_2_, ozone (O_3_) and particulate matter (PM_2.5_) concentrations. Finally, we used monthly averages to calculate ecologically relevant bioclimatic and air quality variables, such as temperature, maximum temperature during the growing season, relative humidity during the growing season, and NO_2_, O_3_ and PM_2.5_ concentrations, by averaging the extracted monthly records over the 5 years. For detailed descriptions of each of the predictors, see Table S1.

Finally, for each tree, we determined the level of sunlight exposure, leaf litter cover and spatial connectivity. Sunlight exposure was estimated according to three categories: (i) tree completely freestanding and unshaded, with the nearest tree or other structure being at least 5 m away from the crown edge, (ii) environment almost open, including those trees less than 25% shaded, and (iii) environment partially shaded, including trees being shaded between 25 and 75% (Nilsson 2007). The percentage ground covered by leaf litter was estimated within a circle around the tree trunk with a 5 m radius (Barr et al. 2021). Host connectivity (e.g., whether trees grow in isolation or are surrounded by many other oaks) for each tree was determined by a visual estimation of the number of neighboring *Q. robur* trees within an observable radius of up to 100 m around the sampled tree (Barr et al. 2021). Within the radius, we counted the number of surrounding trees within two zones (1–50 m and 51–100 m). The connectivity index was then calculated according to the following formula:

$${\text{Connectivity}}\,{\text{index}} = 1 \times \left( {{\text{number}}\,{\text{of}}\, 1{-}50\,{\text{m}}\,{\text{trees}}} \right) + 0.5 \times \left( {{\text{number}}\,{\text{of}}\,51{-}100\,{\text{m}}\,{\text{trees}}} \right)$$

### Statistical analyzes

All analyzes were conducted in R version 4.2.0. For the univariate response variables species richness, species evenness and relative abundance of functional guilds, we fitted linear models using the function *lm* in base R. For the multivariate response community composition, we used the function *adonis2* with a term *by* = *margin* in the *vegan* package (Oksanen et al. 2015; R Core Team 2022). We performed forward selection to guide the identification of the most relevant variables. We started with the null model, i.e., with no explanatory variables. The explanatory variables were tested one by one and selected for inclusion in the regression analysis only if they were statistically significant (*p* < 0.05) using the *stepAIC* function in the *MASS* package (Venables and Ripley 2013). For multivariate analysis, we used function *ordistep* from the *vegan* package to perform forward selection (Oksanen et al. 2022). For each univariate model, we used function *plot_residuals* in the package sjPlot to assess model fit (Lüdecke 2020). We square root-transformed the relative abundance of endophytes to achieve normality of residuals. We assessed multicollinearity in the final models using the variance inflation factor (all VIF < 2; Zuur et al. 2009). We used package *ggplot2* to generate visualizations (Wickham 2009).

To explore the effects of local climate, air quality, habitat factors and host connectivity on the foliar fungal community, we examined fungal richness, evenness, community composition, and the relative abundance of functional guilds in relation to several factors, including average temperature, maximum temperature during the growing season, relative humidity during the growing season, NO_2_, O_3_ and particulate matter concentrations, sunlight exposure (modeled as a categorical variable), leaf litter cover, and host connectivity. After conducting the community composition analysis, we used the *manyglm* function from the *mvabund* package to explore which ASVs differed in their response to significant predictor variables (i.e., maximum temperature during the growing season, NO_2_ concentration and host connectivity) (Wang et al. 2012). Models were fit using a negative binomial error distribution and a log link function. We conducted this analysis for the twenty most abundant fungal families.

## Results

**Fig. 2:**
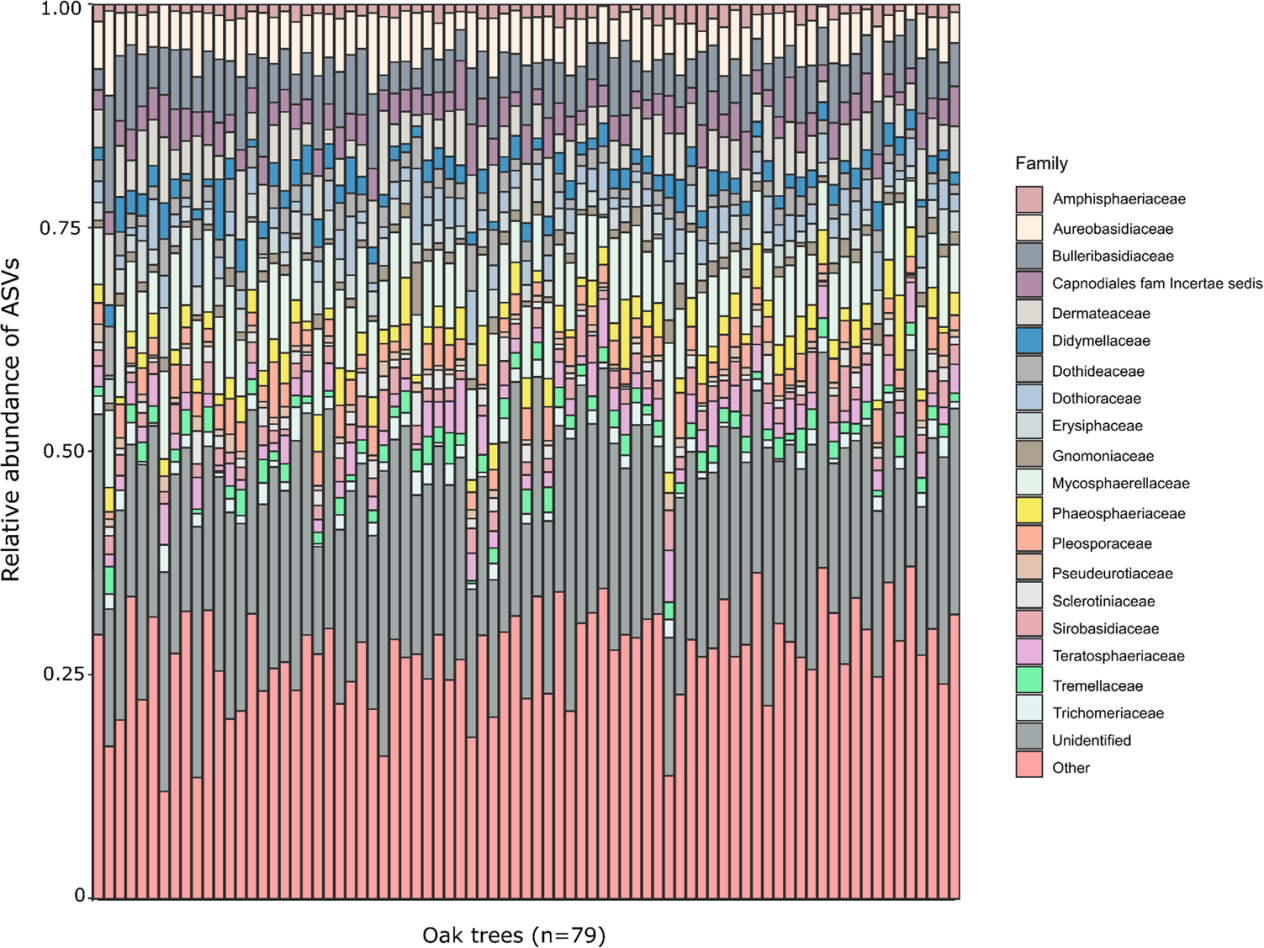
Stacked bar charts showing the relative abundance of fungal families in leaves of 79 pedunculate oak trees (*Quercus robur*). Families with low relative abundance (< 5%) were merged under the category “Other,” while the category “Unidentified” represents taxa for which a putative taxonomic classification is unknown

We detected a total of 5092 fungal ASVs across the 79 trees. The most abundant fungal families were Aureobasidiaceae (23.1%), Mycosphaerellaceae (22.0%), Erysiphaceae (21.9%), Bulleribasidiaceae (16.5%) and Dermateaceae (8.6%) (Fig. 2 and Supplementary Data).

**Fig. 3:**
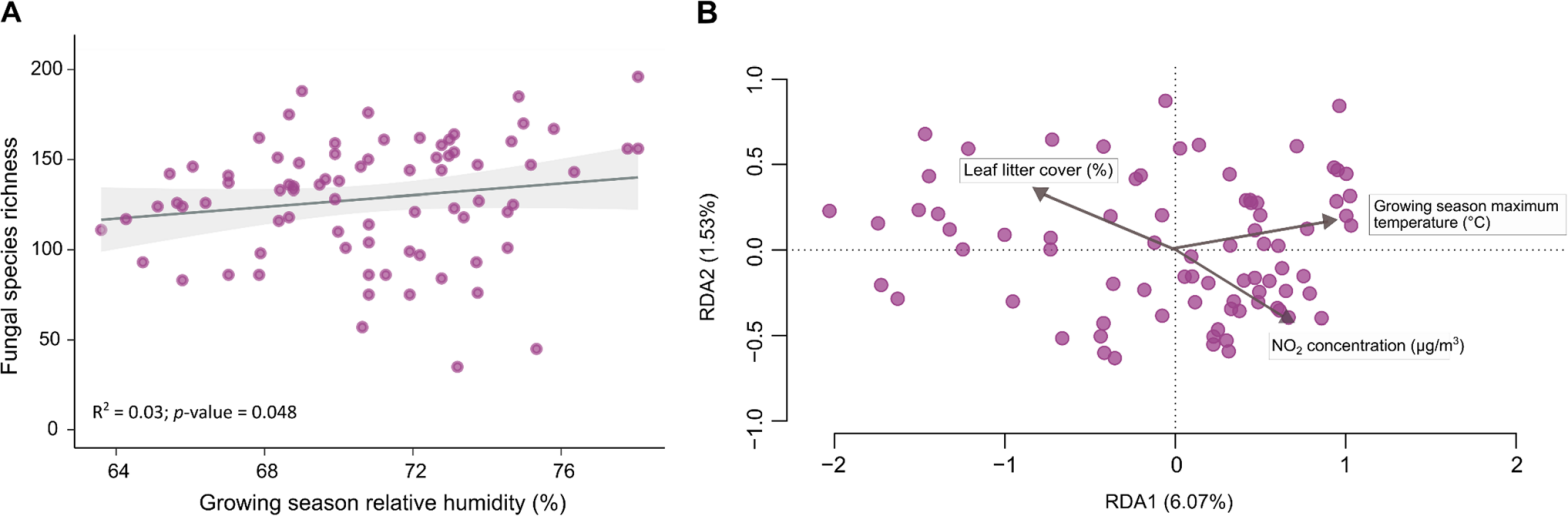
The relationship between fungal richness, community composition, climate, air quality and leaf litter cover. **A** The relationship between fungal richness and growing season relative humidity. Dots represent raw data at the tree-level. **B** A partial canonical redundancy analysis (partial RDA) ordination plot that demonstrates the relative contribution of growing season maximum temperature, NO_2_ concentration and leaf litter cover to fungal community composition. The plot shows the optimal model obtained with *ordistep* in the *vegan* package. Each point represents a foliar fungal community found within a single tree-level sample, while vectors show the main environmental drivers

Fungal species richness was positively related to relative humidity during the growing season (*t*_1,77_ = 1.95, *p* = 0.048, R^2^ = 0.03; Fig. 3a), but there was no significant association between fungal richness and average temperature, maximum temperature during the growing season, air quality, local habitat variables, or host connectivity (Table S4). We did not detect a significant association between fungal evenness and local climate, air quality, local habitat variables, or host connectivity (Table S4). We detected a significant association between fungal community composition and growing season maximum temperature (*F*_1,77_ = 2.46, *p* = 0.002), NO_2_ concentration (*F*_1,77_ = 1.79, *p* = 0.010) and leaf litter cover (*F*_1,77_ = 1.91, *p* = 0.003; Fig. 3b and Table S4). Each of these factors explained 2–3% of variation in fungal community composition.

**Fig. 4:**
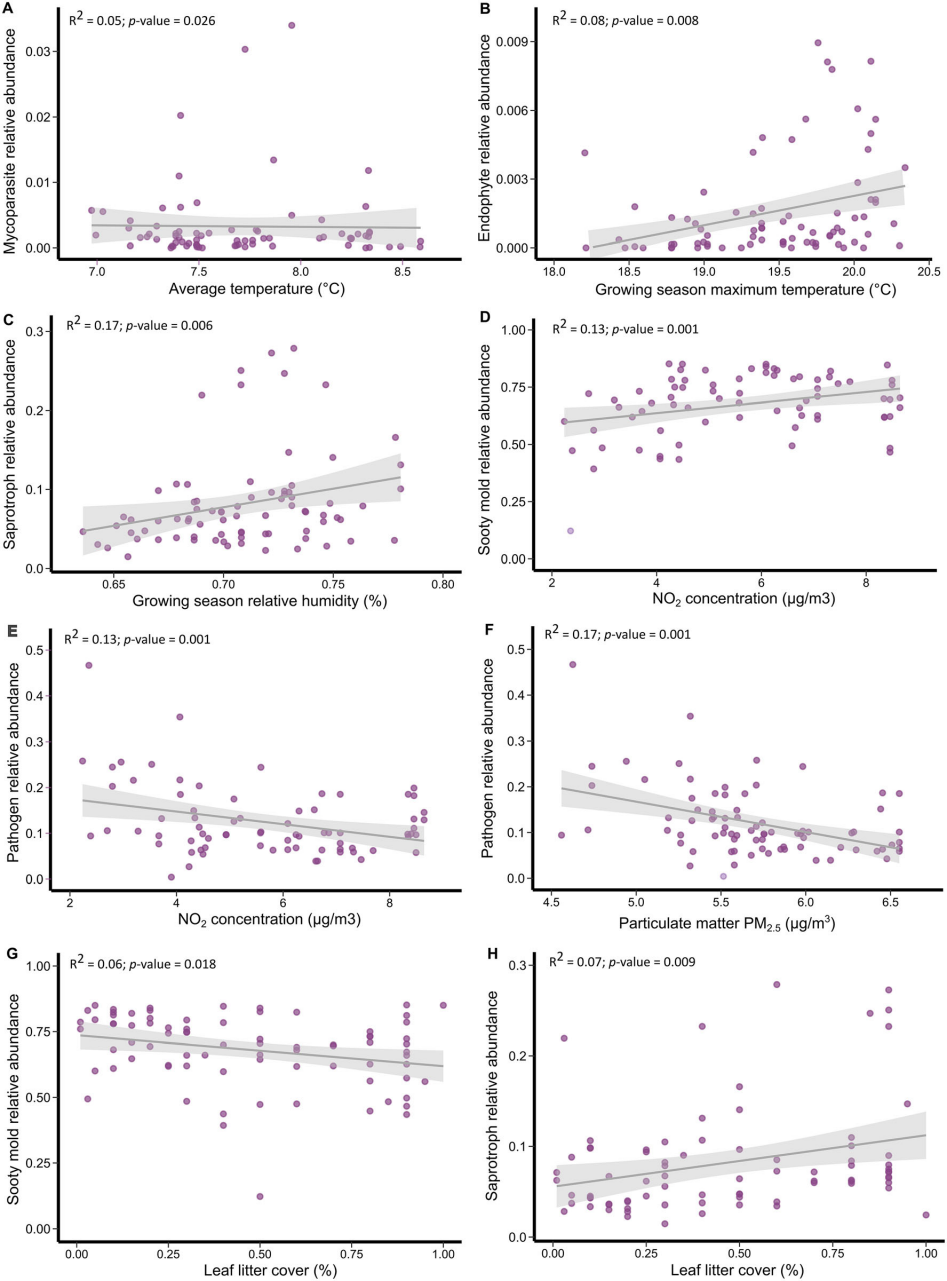
Impact of climate, air quality and leaf litter cover on the relative abundance of fungal guilds. Dots represent raw data at the tree-level

**Fig. 5:**
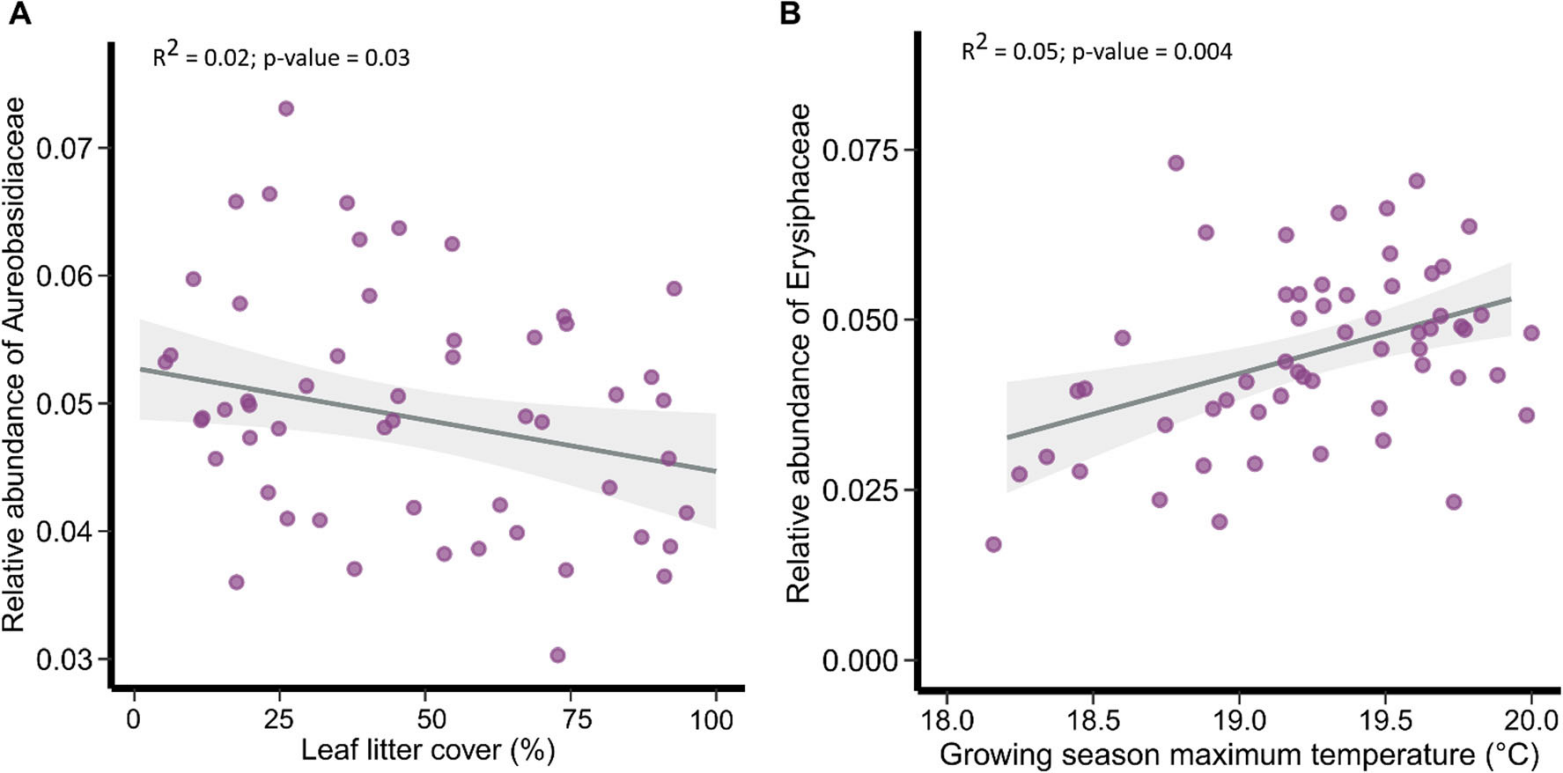
The effect of leaf litter cover (**a**) and growing season maximum temperature (**b**) on the relative abundance of fungal families *Aureobasidiaceae* and *Erysiphaceae*, respectively. Dots represent raw data at the tree-level

The relative abundance of mycoparasites slightly increased with average temperature (*t*_1,77_ = − 2.28, *p* = 0.026; Fig. 4a; Table S5), while fungal endophytes increased with growing season maximum temperature (*t*_1,77_ = 2.92, *p* = 0.005; Fig. 4b). Saprotrophic fungi increased with growing season relative humidity (*t*_1,77_ = 2.05, *p* = 0.043; Fig. 4c), and the relative abundance of sooty molds was higher on trees growing in locations with high NO_2_ concentration (*t*_1,77_ = 3.22, *p* = 0.035; Fig. 4d). Interestingly, the relative abundance of pathogens decreased with higher NO_2_ and PM_2.5_ concentrations (*t*_1,77_ = 2.10, *p* = 0.035 and *t*_1,77_ = 2.33, *p* = 0.022, respectively; Fig. 4e, f). As leaf litter cover increased, sooty molds slightly decreased in their relative abundance, while saprotrophic fungi increased (*t*_1,77_ = − 2.13, *p* = 0.046 and *t*_1,77_ = 2.31, *p* = 0.024, respectively; Fig. 4g, h). Other fungi, from less abundant guilds, such as animal and lichen parasites, lichenised fungi and epiphytes, were not significantly affected by local climate, air quality, local habitat variables, or host connectivity (Table S5). Similarly, there was no significant association between local climate, air quality, local habitat variables, or host connectivity and fungi from category “Unknown” (Table S5). Out of the twenty most abundant fungal families, only two families showed a significant association with the variables studied: The family Aureobasidiaceae decreased with leaf litter cover (Dev = 5.2, *p* = 0.03; Fig. 5a), while the family Erysiphaceae increased with growing season maximum temperature (Dev = 13.9, *p* = 0.004; Fig. 5b).

## Discussion

Our study explored spatial variation in foliar fungal community structure in an urban environment, combining detailed climate and air quality maps with molecular screening of leaf fungi on oak trees in Stockholm. First and foremost, we showed that the richness of fungal species was significantly higher in locations with higher growing season relative humidity, emphasizing the important role of urban climate in shaping fungal diversity. Furthermore, we uncovered that growing season maximum temperature, nitrogen dioxide concentration, and leaf litter cover affected fungal community composition. Finally, our study highlighted the diverse responses of different fungal guilds to urban climate and air quality: mycoparasites slightly preferred locations with higher average temperature, fungal endophytes favored warmer growing season maximum temperatures, and the relative abundance of fungal pathogens was lowest in areas with elevated NO_2_ and PM_2.5_ concentrations. Local habitat factors also played a key role, with sooty molds declining in areas with increased leaf litter cover, while saprotrophic fungi increased in their relative abundance. Certain fungal guilds, including animal and lichen parasites, lichenized fungi, and epiphytes, were unaffected by variation in climate and air quality. Among the most abundant fungal families, we observed that the *Aureobasidiaceae* family decreased in abundance as leaf litter cover increased, whereas the *Erysiphaceae* family thrived in locations with higher growing season maximum temperatures. These findings demonstrate the profound impact of urban climate, air quality, and local habitat factors on the foliar fungal community in urban settings, with significant implications for understanding plant health and ecosystem services.

The finding that fungal species richness showed a positive association with relative humidity during the growing season in our study corroborates with the patterns found in other studies. For example, studies in natural ecosystems, such as forests, have consistently indicated that higher humidity is correlated with increased fungal richness and abundance (Talley et al. 2002). This finding is in line with our predictions, since many fungal groups require moisture for spore germination and growth (Gottlieb 1950). Notably, the relationship between saprotrophic fungi and growing season relative humidity was particularly strong, as demonstrated by their increase in relative abundance. Interestingly, none of the other urban climatic factors, including average temperature and maximum temperature during the growing season, were found to affect fungal richness on oaks. As for fungal community composition, we found that growing season maximum temperature was the main predictor of the variation in community composition among trees. In line with this finding, several observational studies have reported shifts in the composition of foliar fungal communities with temperature (Bálint et al. 2015; Faticov et al. 2021). This finding is especially important for urban landscapes, which are known for their heat islands. Heat islands occur when urban impervious surfaces absorb more sunlight than the surrounding vegetation, which can have pronounced effects on the local climate, for example by increasing air temperatures. This, in turn, affects the fungal communities inhabiting urban trees (Taha 1997; Schwaab et al. 2021). Interestingly, the finding that the relative abundance of fungal endophytes increased with higher average temperatures, while mycoparasites decreased, suggests that temperature plays an important role in shaping the trophic structure of fungal communities on urban trees. These contrasting responses can be explained by different ecological strategies; endophytes are more likely to occupy niches within the leaf tissue, sheltering them from desiccation, while mycoparasites may be more prevalent on the leaf surface, exposing them to drought, UV radiation, and other environmental stressors (Chaudhary et al. 2022; Muthu Narayanan et al. 2022). In summary, our findings not only align with the previous research demonstrating a strong relationship between humidity and fungal richness, but also emphasize the critical role of temperature in shaping fungal communities on urban trees. Enhancing urban tree canopy coverage in cities can effectively reduce temperatures and increase humidity levels. Such measures can maintain or increase fungal richness and diversity, which play an important role in nutrient cycling, organic matter decomposition and disease resistance in trees, thereby improving urban tree health and ecosystem functioning.

Although we did not find a significant correlation between fungal richness and evenness and the concentration of NO_2_, O_3_, and particulate matter (PM_2.5_), our study demonstrated a significant association between fungal community composition and NO_2_ concentration in the atmosphere, as well as between specific fungal guilds and NO_2_ and particulate matter (PM_2.5_) concentrations. There are several plausible mechanisms that may explain this finding. First, NO_2_ was shown to inhibit the growth of some microorganisms, including fungi, and has even been used commercially to sterilize medical devices (Shank et al. 1962; Shomali et al. 2015; Gosling et al. 2016). Second, NO_2_ can be phytotoxic by altering tree physiology (e.g., leaf cuticle properties or the production of antimicrobial compounds) and thereby indirectly impact fungal communities (Siegwolf et al. 2022). Third, NO_2_ and particulate matter (PM_2.5_) can provoke stress responses in plants, potentially making them more susceptible to fungal infections. Weakened host defenses can create a more favorable environment for fungal pathogens to establish and grow. Interestingly, our findings indicate that high NO_2_ and PM_2.5_ concentrations correlate with a decrease in relative abundance of fungal pathogens and increase in relative abundance of sooty molds. At this stage, it is unclear which processes are behind these results, which emphasizes the need for further research to understand the mechanisms and implications of elevated NO_2_ concentrations for foliar fungal communities and tree health.

Among local habitat factors, leaf litter cover was the only factor that explained variation in fungal community composition, whereas it did not significantly affect fungal richness and evenness. The variation in fungal community composition could be explained by a decrease in relative abundance of sooty molds and increase of saprotrophic fungi with higher leaf litter cover. The increase of saprotrophic fungi with leaf litter cover was expected, as these decomposers are known to thrive in environments with high organic matter, where they are essential for ecosystem health through their role in breaking down leaf litter and recycling nutrients (Kubartová et al. 2009). Interestingly, the early stages of leaf decomposition are primarily driven by fungi already present on the living leaves (Voříšková and Baldrian 2013). This suggests that certain fungal species are adapted to different environmental conditions as they actively disperse from living leaves to leaf litter or await leaf fall in autumn. Unlike leaf litter cover, sunlight exposure did not affect fungal richness, evenness, community composition and relative abundance of fungal guilds. This may suggest that climatic heterogeneity may overshadow the effect of sunlight on fungal communities. Similarly, we did not detect a significant association between host connectivity and fungal richness, evenness, community composition and relative abundance of fungal guilds. This finding may suggest a lack of dispersal limitation for foliar fungi, a conclusion reached in several previous studies, though at smaller spatial scales (Cordier et al. 2012; Faticov et al. 2023). As one future research direction, it would be interesting to explore other biotic and abiotic spatial components of the environment, such as the diversity and composition of the neighboring plant community and urban structures. In summary, our findings highlight that leaf litter cover is a critical local habitat factor that can shape the saprotrophic fungi that play important role in organic matter decomposition. To maintain the diversity and abundance of fungi in urban environments, urban planners can designate areas for selective leaf litter retention or establish community composting programs to provide substrates for fungi. Overall, recognizing leaf litter as an important habitat for fungi can aid in designing urban green spaces in a way that enhances their ecological functions.

## Conclusions

Our interdisciplinary approach, combining meteorological insights with microbiological analyzes, has demonstrated an interplay between environmental factors and foliar fungal communities in urban settings. Our study shows an impact of local climate, air quality, and leaf litter cover on the richness, community composition and functional roles of foliar fungi in urban environment. We demonstrated an important role of urban climate, specifically the influence of humidity and temperature, in shaping fungal richness and community composition. Furthermore, we elucidated the complex associations between fungal community composition and environmental factors such as NO_2_ concentration in the air and leaf litter cover, revealing the diverse responses of different fungal guilds. Local habitat factors, particularly leaf litter cover, emerged as an important driver of foliar fungal community in urban tree environments. These findings have significant implications, particularly in urban contexts where heat islands can alter climatic conditions and fungal communities. Overall, we hope that our findings will stimulate future research directions. As one direction, our evidence of the effect of air pollutants on fungal functional guilds opens up the possibility to experimentally study the underlying mechanisms by combining whole metagenome sequencing with the annotation of metabolic pathways of foliar fungi to better understand the responses of fungi with different ecophysiological traits to air pollution. As another important direction, one may conduct similar studies that extend sampling of climate, air quality and the fungal community composition throughout the growing season, which will allow us to generalize patterns across the growing season and examine the time window when climate and air quality matter. Taken together, our research emphasized the need for further interdisciplinary research to decipher the relationships between climate, air quality, and fungal communities and their consequences for the management of urban trees.

## Supplementary Information

Below is the link to the electronic supplementary material.

Supplementary file1 (XLSX 302 kb)

Supplementary file2 (PDF 296 kb)

## Data Availability

Scripts for statistical analyzes, along with taxonomy tables, and metadata used for the analysis are available on FigShare (10.6084/m9.figshare.25325737). All amplicon sequencing data generated in this study is deposited on the National Center for Biotechnology Information’s (NCBI) Sequence Read Archive under BioProject accession number PRJNA1111531 and are available here: http://www.ncbi.nlm.nih.gov/bioproject/1111531.
