# Supplementary material for: Local climate, air quality and leaf litter cover shape foliar fungal communities on an urban tree
Source: Ambio. 2024 Jun 13;53(11):1673–85. doi: 10.1007/s13280-024-02041-4 (PMC11436615; doi:10.1007/s13280-024-02041-4)
Supplement: Supplementary file 2 — Supplementary file2 (PDF 296 kb) [file 13280_2024_2041_MOESM2_ESM.pdf]

Ambio

Supplementary Information

This supplementary information has not been peer reviewed.

Title: Local climate, air quality and leaf litter cover shape foliar fungal communities on an urban tree

Authors: Maria Faticov[1,2*], Jorge H. Amorim[3], Ahmed Abdelfattah[4], Laura J. A. van Dijk[1], Ana Cristina Carvalho[3], Isabelle Laforest-Lapointe[2] and Ayco J. M. Tack[1]

1 Department of Ecology, Environment and Plant Sciences, Stockholm University, Stockholm, Sweden

2 Département de biologie, Université de Sherbrooke, Sherbrooke, Canada, QC

3 Swedish Meteorological and Hydrological Institute (SMHI), Norrköping, Sweden

4 Leibniz Institute for Agricultural Engineering and Bioeconomy (ATB), Potsdam, Germany

[*] Corresponding author; e-mail: maria.faticov@gmail.com

Details on molecular analysis

To characterize the foliar and soil fungal community, we used primers targeting the internal transcribed spacer (ITS2) region (Schoch et al. 2012). We used forward primer fITS7 (Ihrmark et al. 2012) and reverse primer ITS4 (White et al. 1990), which target a 250-450 bp fragment encompassing the entire ITS2. We used a two-step PCR approach to amplify the ITS2 region from each sample. First and second PCR steps were done once for each sample. We included one negative control (i.e. wells containing nothing but PCR reagents) in each amplicon pool. In short, the first PCR reaction mixture consisted of 12.5 µL Kapa HiFi Mastermix (Kapa Biosystems, Woburn, MA, USA), 8.5 µL H2O, 1 µL of each primer (10 nmol/µL), and 2 µL of DNA template. Cycling conditions were 95°C for 5 min, 98°C for 1 min, 36 cycles of 98°C for 40 sec, 58°C for 40 sec, and 72°C for 15 sec, followed by a final elongation step of 72°C for 5 min. The primers for the first PCR reaction consisted of adaptor + primer, giving the forward primer 5'– ACACTCTTTCCCTACACGACGCTCTTCCGATCTGTGARTCATCGAATCTTTG – 3' and reverse primer 5'– AGACGTGTGCTCTTCCGATCTTCCTCSSCTTATTGATATGC – 3'. In the second PCR step 15 µL PCR template, 20 µL Kapa HiFi Mastermix and 2.5 µL of each primer (10 nmol/µL) were used. The primers for the second PCR reaction consisted of Illumina handle + X8 + adaptor, with X8 denoting barcode with unique tags of 8 bp. Reaction conditions for the second PCR were as described above, but with 14 cycles instead of 25. Concentrations were measured using the Qubit dsDNA HS assay kit (Life Technologies), after which samples were pooled in equal concentrations. To test whether potential fungal DNA was amplified, we ran the PCR products on an Agilent 2100 Bioanalyzer (Agilent, Santa Clara, CA, USA). The initial bioinformatic processing was done in the DADA2 pipeline following the standard protocol (Callahan et al. 2016). In short, we eliminated reads with quality scores less than 10 (truncQ = 10). We also performed consensus chimera

removal, where chimeras were identified in each sample independently. Paired reads were assembled using the mergePairs function with a minimum overlap of 20 bp and allowing a maximum mismatch of 5% within the region of overlap.

Table S1. Climatic, air quality, local habitat and host connectivity factors and their hypothesised influence on the foliar fungal community.

Predictor	Description	Predictions
Average temperature (°C)	Monthly temperature averaged across 5 years	Fungal species richness and evenness increase with higher average temperature.<br><br>Average temperature explains the differences in foliar fungal community composition among trees.<br><br>We expected to detect a significant association between relative abundance of sooty molds, endophytes, saprotrophs, pathogens, mycoparasites and average temperature. We predicted that the strongest association will be for sooty molds, saprotrophs, and pathogens.
Growing season maximum temperature (°C)	Monthly max. temperature averaged across 5 years for the period of May-August	Fungal species richness and evenness increase with higher growing season maximum temperature.<br><br>Growing season maximum temperature explains the differences in foliar fungal community composition among trees.<br><br>We expected to detect a significant association between relative abundance of sooty molds, endophytes, saprotrophs, pathogens, mycoparasites and growing season maximum temperature. We predicted that the strongest association will be for sooty molds, saprotrophs, and pathogens.
Growing season relative humidity (%)	Monthly relative humidity averaged across 5 years for the period of May-August	Fungal species richness and evenness increase with higher growing season relative humidity.<br><br>Growing season relative humidity explains the differences in foliar fungal community composition among trees.<br><br>We expected to detect a significant association between relative abundance of sooty molds, endophytes, saprotrophs,

Predictor	Description	Predictions
		pathogens, mycoparasites and growing season relative humidity. We predicted that the strongest association will be for sooty molds, saprotrophs, and pathogens.
NO_2 concentration (μg/m^3)	Monthly NO_2 concentration averaged across 5 years	Fungal species richness and evenness decrease with higher NO_2 concentration.<br><br>NO_2 concentration explains the differences in foliar fungal community composition among trees.<br><br>We expected that higher NO_2 concentration would be associated with the lower relative abundance of sooty molds, saprotrophs, pathogens, mycoparasites, but not endophytes.
O_3 concentration (μg/m^3)	Monthly O_3 concentration averaged across 5 years	Fungal species richness and evenness decrease with higher O_3 concentration.<br><br>O_3 concentration explains the differences in foliar fungal community composition among trees.<br><br>We expected that higher O_3 concentration would be associated with the lower relative abundance of sooty molds, saprotrophs, pathogens, mycoparasites, but not endophytes.
Particulate matter $PM_{2.5}$ (μg/m^3)	Monthly $PM_{2.5}$ concentration averaged across 5 years	Fungal species richness and evenness decrease with higher $PM_{2.5}$ concentration.<br><br>Particulate matter $PM_{2.5}$ explain the differences in foliar fungal community composition among trees.<br><br>We expected that higher concentration of particulate matter ($PM_{2.5}$) would be associated with the lower relative abundance of sooty molds, saprotrophs, pathogens, mycoparasites, but not endophytes.

Predictor	Description	Predictions
Leaf litter cover (%)	Percentage of ground cover within a 5 m radius of the sampled tree	Fungal species richness and evenness increase with the higher percentage of leaf litter cover.<br><br>Leaf litter cover explains the differences in foliar fungal community composition among trees.<br><br>We expected to detect a significant association between relative abundance of sooty molds, saprotrophs, pathogens, mycoparasites and leaf litter cover. We predicted that the strongest association will be for sooty molds, saprotrophs, and pathogens, given that the members of these fungal groups may overwinter in leaf litter.
Sunlight exposure	Freestanding, less than 25% shaded or 25–75% shaded tree	Fungal species richness and evenness are the highest on shaded trees.<br><br>Sunlight exposure explains the differences in foliar fungal community composition among trees.
Host connectivity	Calculated as: 1 × (number of trees within 1–50 m) + 0.5 × (number of trees within 51–100 m))	Regarding host connectivity, we expected that oak trees growing closer to neighboring oaks would have higher species richness and evenness due to facilitation of microorganism dispersal among nearby trees.<br><br>Host connectivity explains the differences in foliar fungal community composition among trees.

Table S2. Definitions of functional guilds used for fungal taxa (assigned using *FungalTraits* database) *

FUNGAL GUILDS ASSIGNED TO LEAF-ASSOCIATED TAXA	
**Sooty mold**	Fungi in the groups *Aureobasidium* and *Scorias*
**Endophytes**	Fungi that inhabit plant tissues as endophytes (asymptomatic, commensal or weakly mutualistic species)
**Saprotrophs**	Fungal saprotrophs, including fungi that degrade wood, litter, dung, nectar, pollen and unspecified substrates
**Pathogens**	Fungal pathogens that penetrate the leaf tissues and cause visible symptoms
**Mycoparasites**	Fungi that can parasitize other fungi
**Other**	Lichenized fungi, animal and lichen parasites and a few epiphytes (which are known to live on plant surface) from the genera *Stomiopeltis*, *Buckleyzyma*, *Symmetrospora* and *Camptophora*
**Unknown**	Fungi from phylum Ascomycota and Basidiomycota, which were not assigned to any taxa or which functions are unknown

**Functional guilds were assigned using FungalTraits database described in* (Põlme et al. 2020).

Table S3. Monthly mean air temperature and precipitation anomalies between selected years and WMO´s 1961-1990 climate normals. Data provenance: Stockholm-Observatoriekullen weather station, available at https://www.smhi.se/klimat.

	Monthly mean air temperature (ºC) / Precipitation (mm) anomalies					
**Year**	**Annual**	**May**	**June**	**July**	**Aug.**	**Growing season**
**2006**	1.8/10	0.9/16	1.6/-13	3.6/-39	2.9/80	2.3/11
**2007**	1.5/-37	0.9/-5	1.2/22	-0.2/-21	1.5/-49	0.9/-13
**2012**	0.6/240	1.7/-10	-1.7/115	0.5/-11	0.5/55	0.3/37
**2013**	1.2/-80	2.6/-13	1.1/1	1.2/-29	1.5/-11	1.6/-13
**2014**	2.2/75	0.6/5	-1.1/-17	3.5/-28	1.1/49	1.0/2

Table S4. The relationship between local climate, air quality, habitat factors and host connectivity and fungal richness, evenness and community composition. In the final univariate models (sections a and b), the model outputs presented are t-values, P-values, standardized regression coefficients, standard errors, and adjusted coefficient of determination (R^2). As for the multivariate models (section c), the model output includes F-values, P-values, sums of squares (SS), and partial coefficient of determination (R^2).

**a. Fungal richness**	**t-value**	***P*-value**	**b**	**SE (b)**	**R^2 (%)**
Final model: Fungal richness ~ Relative humidity during growing season					
Intercept	-0.31	0.757	-24.55	33.43	
Growing season relative humidity	1.95	0.048	0.01	0.02	3
**b. Fungal evenness**					
Final model: Intercept only					
**c. Fungal community composition**	**F-value**	***P*-value**	**SS**	**df**	**R^2 (%)**
Final model: Fungal community composition ~ Growing season maximum temperature + NO_2 concentration + Leaf litter cover					
Growing season maximum temperature	2.46	**0.002**	0.45	1	2.9
NO_2 concentration	1.79	**0.010**	0.33	1	2.2
Leaf litter cover	1.91	**0.003**	0.35	1	2.3

Table S5. The relationship between local climate, air quality, habitat factors and host connectivity and fungal functional guilds. Shown are t-values, P-values, standardized regression coefficients, standard errors, and adjusted coefficient of determination (R^2).

**a. Sooty mold**		**t-value**	***P*-value**	**b**	**SE (b)**	**R^2 (%)**
Final model: Relative abundance of sooty molds ~ Leaf litter cover + NO_2 concentration						
	Intercept	12.21	**< 0.001**	0.60	0.05	16
	Leaf litter cover	-2.13	**0.046**	-0.10	0.05	
	NO_2 concentration	3.22	**0.002**	0.02	0.01	
**b. Pathogens**						
Final model: Pathogens ~ Particulate matter ($PM_{2.5}$) + NO_2 concentration						
	Intercept	0.93	0.356	0.16	0.17	17
	Particulate matter ($PM_{2.5}$)	2.33	**0.022**	0.08	0.03	
	NO_2 concentration	2.10	**0.035**	0.02	0.01	
**c. Saprotrophs**						
Final model: Saprotrophs ~ Leaf litter cover + Growing season relative humidity						
	Intercept	-1.63	0.107	-0.22	0.13	11
	Leaf litter cover	2.31	**0.024**	0.05	0.02	
	Growing season relative humidity	2.05	**0.043**	0.39	019	
**d. Endophytes**						
Final model: Endophytes ~ Growing season maximum temperature						
	Intercept	-2.90	**0.005**	-0.37	0.13	9
	Growing season maximum temperature	2.92	**0.005**	0.001	0.001	
**e. Mycoparasites**						
Final model: Mycoparasites ~ Average temperature						
	Intercept	2.29	0.025	6.21	2.71	5
	Average temperature	-2.28	**0.026**	-0.02	0.01	
**f. Other**						
Final model: Intercept only						
**g. Unknown**						
Final model: Intercept only						

References

Ihrmark, K., I. T. M. Bödeker, K. Cruz-Martinez, H. Friberg, A. Kubartova, J. Schenck, Y. Strid, J. Stenlid, et al. 2012. New primers to amplify the fungal ITS2 region--evaluation by 454-sequencing of artificial and natural communities. *FEMS microbiology ecology* 82: 666–677. doi:10.1111/j.1574-6941.2012.01437.x.

Põlme, S., K. Abarenkov, R. Henrik Nilsson, B. D. Lindahl, K. E. Clemmensen, H. Kauserud, N. Nguyen, R. Kjøller, et al. 2020. FungalTraits: a user-friendly traits database of fungi and fungus-like stramenopiles. *Fungal Diversity* 105: 1–16. doi:10.1007/s13225-020-00466-2.

Schoch, C. L., K. A. Seifert, S. Huhndorf, V. Robert, J. L. Spouge, C. A. Levesque, W. Chen, and F. B. Consortium. 2012. Nuclear ribosomal internal transcribed spacer (ITS) region as a universal DNA barcode marker for fungi. *Proceedings of the National Academy of Sciences* 109: 6241–6246. doi:10.1073/pnas.1117018109.

White, T. J., T. Bruns, S. Lee, and J. Taylor. 1990. Amplification and direct sequencing of fungal ribosomal RNA genes for phylogenetics. In *PCR Protocols*, ed. T. J. White, M. A. Innis, D. H. Gelfand, and J. J. Sninsky, 315–322. New York, USA: Elsevier. doi:10.1016/B978-0-12-372180-8.50042-1.
